# Editorial: Interaction effect of low carbohydrate diets and exercise on weight loss and cardio-metabolic health

**DOI:** 10.3389/fnut.2022.1074689

**Published:** 2022-11-09

**Authors:** Fenghua Sun, Zhaowei Kong, Paulo A. S. Armada-da-Silva, Antonio Paoli

**Affiliations:** ^1^Department of Health and Physical Education, The Education University of Hong Kong, Hong Kong, Hong Kong SAR, China; ^2^Faculty of Education, University of Macau, Taipa, Macao SAR, China; ^3^Faculdade de Motricidade Humana, Universidade de Lisboa, Lisbon, Portugal; ^4^Department of Biomedical Sciences, University of Padua, Padua, Italy

**Keywords:** obesity, ketogenic diet, diet-based therapy, high-intensity exercise, non-pharmacological intervention

The worldwide overweight and obesity epidemic has drawn significant attention. The obesity-related disorders, including musculoskeletal disorders, cardiovascular disease, type 2 diabetes (T2D), neurodegenerative diseases, certain types of cancer, and psychological problems, not only affect the quality of life of the overweight and obese population but also impose a high societal and economic burden (1, 2). On the other hand, even a small reduction (e.g., 5%) in the body weight of obese individuals may lead to clinically significant decreases in risk factors for non-communicable diseases. Thus, it would be important and urgent to explore effective and inexpensive strategies for facilitating weight loss and preventing weight gain.

Diet and exercise remain the two most important non-pharmacological interventions for the prevention and treatment of obesity. Given that low carbohydrate diets (LCDs) result in mild hyperketonemia and ketone bodies become a viable alternative fuel source during metabolic stress, LCDs including the ketogenic diet have been used to achieve weight loss and improve cardio-metabolic health, despite a few light adverse effects (e.g., loss of muscle mass) associated with caloric restriction and complex physiological (e.g., sodium and potassium balance) and metabolic (e.g., effects on liver and muscle glycogen storage) alterations (3, 4). Exercise is an effective strategy to stimulate fat utilization in the muscle and cardiovascular systems, as well as to increase the synthesis and release of certain neurotransmitters and neurotrophic factors related to fat metabolism. As a result, exercise such as moderate-intensity continuous training (MICT), high-intensity interval training (HIIT), or high-intensity circuit training (HICT) may positively impact physical and mental health, including body weight control (5, 6). It is reasonable to speculate that the combination of LCDs with exercise may enhance their therapeutic effects. Several pilot studies observed additional improvement in cardiorespiratory fitness when LCDs were combined with extra HIIT or MICT (7–9). Therefore, this Research Topic further extended the previous research and aimed to explore how to combine LCDs and exercise interventions to maximize their potential benefits, including but not limited to body weight reduction, cardiometabolic health, and physiological responses.

In this Research Topic, a total of four papers were published to cover the above-mentioned aspects.

The first study (Sun et al.) evaluated the effects of combining LCDs and different training protocols, i.e., HIIT or MICT, on gut microbiota and cardiometabolic-related profiles among a group of young overweight females. After a 4-week intervention, the Shannon, Chao 1, and Simpson indexes were not changed in both groups. However, compared with the control group, combining LCDs and HIIT/MICT increased the short-chain fatty acids (SCFA)-producing Blautia genus and reduced the T2D-related genus Alistipes. The authors concluded that a 4-week LCDs did not change the α-diversity and overall structure of gut microbiota, whereas combining LCDs with HIIT/HICT may have additional benefits on gut physiology.

The second study (Hu et al.) further explored the benefits of LCDs with or without exercise on mental health, eating behavior, and physiological parameters such as body composition, aerobic fitness, blood pressure, lipid profile, and some metabolic hormones. Three groups of young overweight females completed 4 weeks of interventions, i.e., maintaining their regular diet (CON), taking LCDs only (LC-CON), and taking LCDs together with additional exercise training (LC-EXE). Although eating behaviors were not changed in all three groups, significant body weight reduction and decreases in certain metabolic hormones such as insulin, C-peptide, and leptin were observed in both LCDs groups. Importantly, compared with the LC-CON group, combining LCDs and exercise produced a greater improvement in cardiovascular fitness and anxiety levels.

The third study (Camajani et al.) explored the efficacy of a very low-calorie ketogenic diet (VLCKD) combined with interval training on body weight, body composition, and physical performance in patients with sarcopenic obesity (SO). This topic is also important as the prevalence of SO is increasing worldwide, and it is expected to pose significant challenges to public health, especially considering the aging societies nowadays. This study recruited 24 patients with SO aged between 50 and 70 years and divided them into the VLCKD group and VLCKD with interval training (VLCKD-IT) group. Before and after 6 weeks of interventions, anthropometric indexes, body composition, muscle strength, and physical performance were assessed. The results indicated that both groups showed similar reductions in body mass index, body weight, waist and hip circumferences, and improvements in muscle strength and physical performance. Compared with the VLCKD group, the VLCKD-IT group demonstrated better fat-free mass preservation and greater fat-mass reduction. However, one should be cautious in generalizing these results because of the study limitations, such as the small sample size and the lack of follow-up.

Last, there is one more paper that deserves a mention. Lian et al. performed one animal study to explore the mechanisms behind the high-fat diet (HFD)-induced pain susceptibility. The results demonstrated that defective branched-chain amino acids (BCAAs) catabolism in lumbar dorsal root ganglia (DRG) facilitates HFD-induced pain hypersensitivity by triggering inflammation. These findings may serve as a basis for pathogenesis and offer potential targets for developing diet-based therapy for chronic pain.

In summary, the results of the studies mentioned above explored the potential benefits of LCDs with or without exercise on physical and mental health. Because of the limited number of studies included in this topic, it is difficult to make any strong recommendations, and many aspects are still to be clarified. Therefore, continuing research on this exciting and important topic should be encouraged.

## Author contributions

ZK wrote the introduction and the conclusion. FS wrote the central part with comments on the cited papers and references. All authors contributed to the article and approved the submitted version.

## Conflict of interest

The authors declare that the research was conducted in the absence of any commercial or financial relationships that could be construed as a potential conflict of interest.

## Publisher's note

All claims expressed in this article are solely those of the authors and do not necessarily represent those of their affiliated organizations, or those of the publisher, the editors and the reviewers. Any product that may be evaluated in this article, or claim that may be made by its manufacturer, is not guaranteed or endorsed by the publisher.

## References

[1] Gómez-Ambrosi J Catalán V Rodríguez A Andrada P Ramírez B Ibáñez P. Increased cardiometabolic risk factors and inflammation in adipose tissue in obese subjects classified as metabolically healthy. Diabetes Care. (2014) 37:2813–21. 10.2337/dc14-0937 25011950

[2] Mariam A Miller-Atkins G Pantalone KM Iyer N Misra-Hebert AD Milinovich A. Associations of weight loss with obesity-related comorbidities in a large integrated health system. Diabetes Obes Metab. (2021) 23:2804–13. 10.1111/dom.14538 34472680 PMC9292723

[3] Ross LJ Byrnes A Hay RL Cawte A Musial JE. Exploring the highs and lows of very low carbohydrate high fat diets on weight loss and diabetes-and cardiovascular disease-related risk markers: a systematic review. Nutr Diet. (2021) 78:41–56. 10.1111/1747-0080.12649 33283417

[4] Drabińska N Wiczkowski W Piskuła MK. Recent advances in the application of a ketogenic diet for obesity management. Trends Food Sci Technol. (2021) 110:28–38. 10.1016/j.tifs.2021.01.080

[5] WHO. Global Recommendations on Physical Activity for Health. Geneva: World Health Organization (2010). 26180873

[6] Cox CE. Role of physical activity for weight loss and weight maintenance. Diabetes Spectr. (2017) 30:157–60. 10.2337/ds17-0013 28848307 PMC5556592

[7] Kong Z Sun S Shi Q Zhang H Tong T Nie J. Short-term ketogenic diet improves abdominal obesity in overweight/obese chinese young females. Front Physiol. (2020) 11:856. 10.3389/fphys.2020.00856 32848830 PMC7399204

[8] Sun S Kong Z Shi Q Hu M Zhang H Zhang D. Non-energy-restricted low-carbohydrate diet combined with exercise intervention improved cardiometabolic health in overweight chinese females. Nutrients. (2019) 11:3051. 10.3390/nu11123051 31847246 PMC6950598

[9] Sun S Kong Z Shi Q Zhang H Lei OK Nie J. Carbohydrate restriction with or without exercise training improves blood pressure and insulin sensitivity in overweight women. Healthcare. (2021) 9:637. 10.3390/healthcare9060637 34072093 PMC8229341

